# A Brief Introduction on Latent Variable Based Ordinal Regression Models With an Application to Survey Data

**DOI:** 10.1002/sim.10208

**Published:** 2024-10-28

**Authors:** Johannes Wieditz, Clemens Miller, Jan Scholand, Marcus Nemeth

**Affiliations:** ^1^ Department of Medical Statistics University Medical Center Göttingen Göttingen Germany; ^2^ Department of Anaesthesiology University Medical Center Göttingen Göttingen Germany; ^3^ Department of Anesthesiology Children's Orthopedic Hospital Aschau im Chiemgau Germany

**Keywords:** cumulative link models, factors influencing willingness to consent to participation, latent variable, logistic regression, ordinal regression, response distribution

## Abstract

The analysis of survey data is a frequently arising issue in clinical trials, particularly when capturing quantities which are difficult to measure. Typical examples are questionnaires about patient's well‐being, pain, or consent to an intervention. In these, data is captured on a discrete scale containing only a limited number of possible answers, from which the respondent has to pick the answer which fits best his/her personal opinion. This data is generally located on an ordinal scale as answers can usually be arranged in an ascending order, for example, “bad”, “neutral”, “good” for well‐being. Since responses are usually stored numerically for data processing purposes, analysis of survey data using ordinary linear regression models are commonly applied. However, assumptions of these models are often not met as linear regression requires a constant variability of the response variable and can yield predictions out of the range of response categories. By using linear models, one only gains insights about the mean response which may affect representativeness. In contrast, ordinal regression models can provide probability estimates for all response categories and yield information about the full response scale beyond the mean. In this work, we provide a concise overview of the fundamentals of latent variable based ordinal models, applications to a real data set, and outline the use of state‐of‐the‐art‐software for this purpose. Moreover, we discuss strengths, limitations and typical pitfalls. This is a companion work to a current vignette‐based structured interview study in pediatric anesthesia.

## 1 Introduction

Data derived from surveys or patient interviews are often subject of research in medicine. As answers are usually encoded numerically, for example, using the numeric rating scale for pain, data analysis using linear models often seems reasonable. Whereas for finely graduated response scales summary statistics such as mean or median response are often of interest, these are often not very meaningful or representative for response scales with only a few categories, for example, “On a scale of 1 (absolutely yes) to 5 (absolutely no), do you consent to the participate in the following study?”. In this case, probabilities for the individual response levels or the proportion of responders who at least “rather consent” to participation would be more revealing. The application of this statistical methodology is illustrated using a vignette‐based interview study from pediatric anesthesia (German Clinical Trials Register DRKS00027090).

### 1.1 Motivation

The setting of the study is as follows: Parents or legal representatives of inpatient children were asked for their willingness to consent to participation in three *hypothetical* studies containing potential objectives for clinical studies in pediatric anesthesia that widely differed in their level of complexity. Aim of this investigation was to identify factors influencing the willingness to consent to participate (acronym filippa) in the following studies:

(1) a prospective *observational study* on a non‐invasive temperature measurement sensor,
(2) a *randomized controlled trial* (RCT) of inducing anesthesia by intravenous vs. inhalative agents, and
(3) a *pharmacological study* on intravenous ibuprofen (painkiller) designed to collect data for regular approval in children, corresponding to an open label phase‐II pharmacological study.

Interviews were conducted by the same investigator to ensure consistent wording and in the same order, with the least complex protocol presented first and the most complex protocol presented last. Legal representatives were asked if they were willing to have their child participating in the corresponding studies. Responses were captured on a five‐point Likert‐scale of “*absolutely consent*”, “*rather consent*”, “*unsure*”, “*rather decline*”, and “*absolutely decline*” participation. For further details on experimental design and the exact descriptions of the studies, we refer to Miller et al. [1].

**FIGURE 1:**
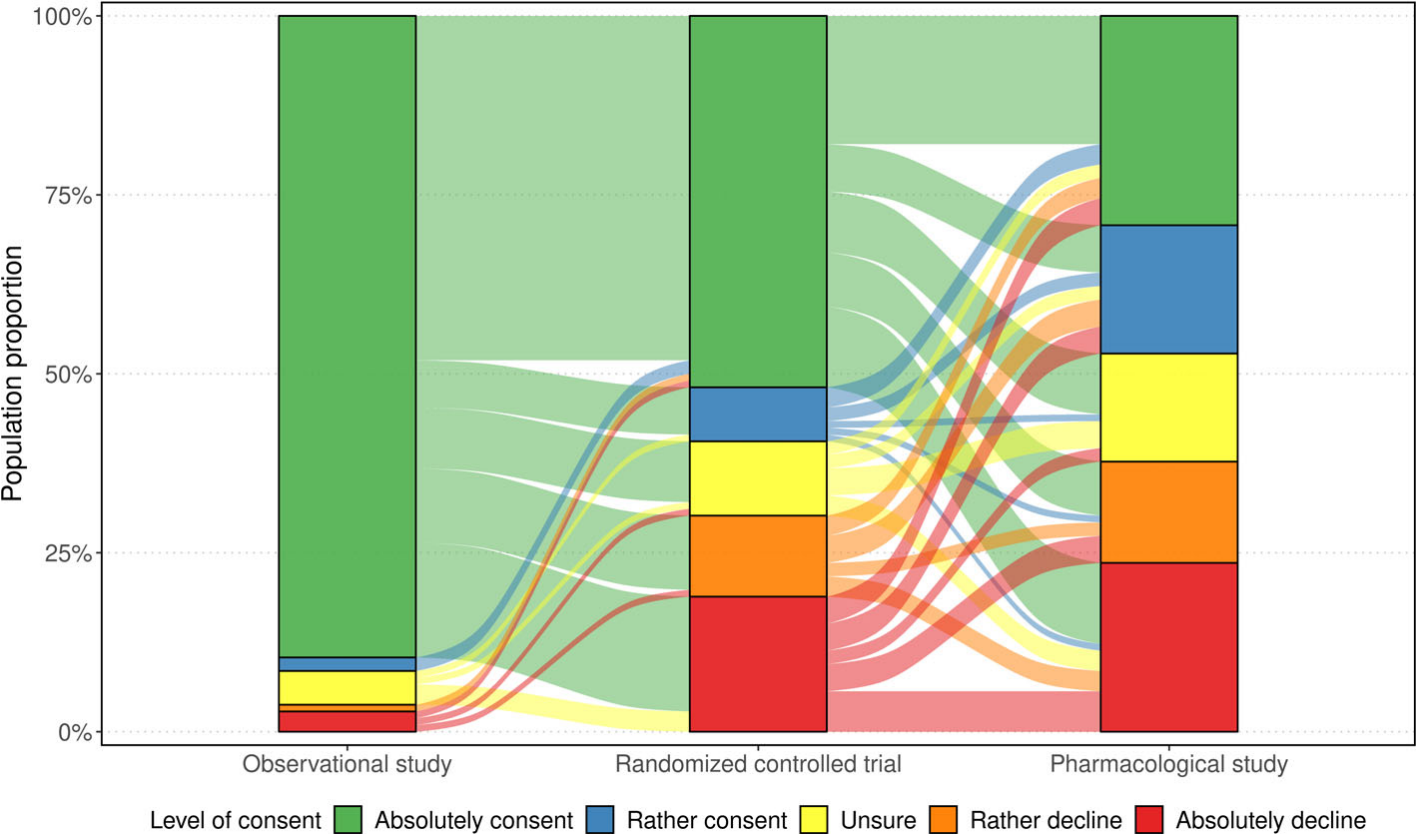
Alluvial diagram of the migration of the level of consent for three hypothetical studies with increasing level of complexity (left to right) indicated by the streams between the bars. The bar charts show the frequency distribution of the corresponding responses for a given study.

Figure 1 portrays a descriptive statistic of the response distribution stratified by the three studies with increasing levels of complexity (bar charts, left to right) in form of an alluvial diagram. The streams between the bar charts show the migration between the answers from one question to the following one. It appears that with an increasing level of complexity, willingness to participate generally decreases. Note that this behavior is, however, not present in all participants. This might be due to the fact that a certain understanding of research in medicine is beneficial to understand the different degrees of invasiveness between the studies, particularly between the RCT and the pharmacological study.

Statistical inference, particularly identification of significantly influencing factors or testing, however, is only possible within a statistical model. For this purpose, we perform an *ordinal regression* on the response depending on the study complexity and including various covariates such as child's sex, age, preceding participation in studies, as well as age and professional medical degree of the legal representatives.

The applied statistical approach presented here is based on the literature briefly summarized below. Agresti [2] provides a comprehensive introduction to categorical data analysis but early work can already be found in Hildebrand, Laing, and Rosenthal [3] and Johnson and Albert [4]. Agresti [5] and McCullagh [6] focus particularly on ordinal models, and Fullerton [7] provides a concise comparison of different types of models. Goodness of fit and model selection problems as well as summary measures of the model's predictive power are addressed in Fagerland and Hosmer [8], Agresti and Kateri [9], and Agresti and Tarantola [10]. Most ordinal regression fall into the category of *generalized linear models*. To this end, Agresti [11] provides a detailed derivation. A software implementation based on these generalized linear models (so‐called *cumulative link models*) as well as a comprehensive how‐to‐apply‐it tutorial is given in Christensen [12, 13]. A clear online introduction using software examples can also be found under Dunn [14, 15]; the latter also covers issues from Bayesian ordinal regression. A broad overview about the topic and the framework including online resources is, moreover, also provided by Harrell Jr. [16, 17, 18, 19, 20]. More recent articles demonstrate the applications of cumulative link models in deep learning classification algorithms, see for example, Vargas, Gutiérrez, and Hervás‐Martínez [21]. Regarding applications in medical research, Norris et al. [22] present a concise comparison between different approaches. For more finely graduated responses scales, such as for example, verbal, visual analogue or numeric scales, Heller, Manuguerra, and Chow [23] present an approach for data analysis. Manuguerra, Heller, and Ma [24] provide corresponding software to do so.

### 1.2 Audience

The target audience of this article is statisticians and medical researchers with a profound quantitative background who have not applied ordinal methods so far. We provide a brief introduction to the fundamentals of latent variable based ordinal models–an approach which yields an easy starting point to the topic in our opinion. This article particularly focuses on the analysis of data on response scales with only few levels and provides practical recommendations, enriched with corresponding R code sections. For additional information such as alternative approaches, software or model extensions we provide references to the literature at appropriate positions.

### 1.3 R Package ordinal

For the analyses within this tutorial we employ the R package ordinal [12] which is available on CRAN, or via https://github.com/runehaubo/ordinal. Code sections are presented at suitable positions and were executed using R version 4.3.2 [25]. The package supports fitting of ordinal fixed effects models (clm) as well as mixed effects models (clmm). To use the package, no extraordinary data preprocessing is required. An overview of the data set is provided below:

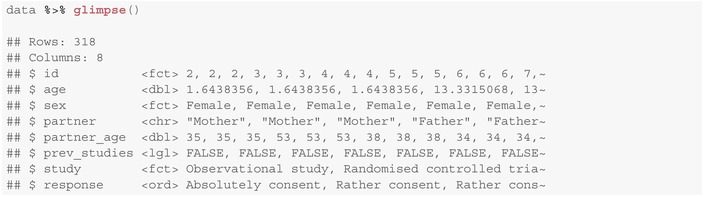

Presented results are based moreover on the packages emmeans, ggstream and tidyverse [26, 27, 28]. Several graphical parameters have been outsourced; code is provided in the Supplement.

### 1.4 Further Reading

There are a number of alternative approaches to ordinal regression and software packages providing tools to this end beyond the latent variable based approach presented here. The introduction of these approaches and software is beyond the scope of this paper. We state some reading recommendations in the following.

Yee [29] present vector generalized linear and additive models requiring only that the regression coefficients enter through a set of linear predictors. The corresponding R package VGAM can be found in Yee [30]. This package is highly flexible and allows, among other things, for multivariate ordinal responses or relaxing the proportional odds assumptions in considering partial proportional odds models.

Moreover, Harrell Jr. [16] provides with the corresponding R package rms [19] a large number of functions for fitting models, estimation, tests, confidence intervals, interpreting and displaying results. To be emphasized should be the functions orm and lrm which are primarily used for continuous and discrete ordinal data, respectively. These functions also implement cumulative link models, but without scale effects, partial or structured thresholds. A Bayesian alternative is implemented in the brms package [31] or by the blrm function of the rmsb package [20] which often promises better convergence behaviors in presence of random effects in the model. For many additional resources for ordinal models, we refer to Harrell Jr. [17, 18].

### 1.5 Outline

This article is structured as follows: Section 2 puts ordinary linear regression and ordinal models in juxtaposition and points out each strengths and limitations. Section 3 introduces the fundamental ideas of latent variable based ordinal regression models. Section 4 considers the influence of discrete and continuous covariates on the response outcome and examine different aspects on estimated parameters and confidence intervals, prediction, goodness of fit and outline numerical issues that may arise. We allude to advanced topics in Section 5 and summarize our findings in Section 6. All computations are stated along with corresponding R code sections. An interactive version of this manuscript can be found at https://jwieditz.github.io/FILIPPA.

## 2 Ordinal Models: Strengths, Limitations and Alternatives

The question of how to evaluate ordinal response data appropriately has been widely discussed in the literature, cf. Agresti [2, chapter 1]. So far, however, no broad consensus has been found as the chosen approach often depends on the original question to be answered.

Approaches considering ordinal responses only as mere categorical data do not exploit the additional structure. In contrast to these nominal approaches, ordinal models can provide descriptive statistics similar to ordinary linear regression, such as means, slopes or correlations. Furthermore, ordinal analysis can use a greater variety of models. These models are usually more efficient yielding higher power for detecting trends or location alternatives using fewer parameters. Moreover, these parameters are often simpler in their interpretation than parameters in standard models for nominal variables, cf. Agresti [2, section 1.2].

For ordinal data with many response levels, as for instance for visual analogue/ numeric scales, ordinal models are often inappropriate as individual response levels are not that meaningful or fitting might even be infeasible if the number of parameters to be estimated grows too large [23]. For this kind of data, responses are commonly already encoded numerically in the questionnaire. As a result, ordinary least squares analysis is usually less problematic and provides more interpretable insights than an ordinal analysis, cf. Agresti [2, section 1.2].

In contrast, for ordinal data consisting of only a few response levels, ordinal regression analysis is often the better choice even though a linear regression analysis can be useful for identifying variables that clearly affect the response variable. Agresti [2, section 1.3.2] points out a number of reasons why ordinary linear regression is in this case often inappropriate:

(a) There is usually *not* a clear‐cut choice for the scores, that is, a particular response outcome might be consistent with a range of values of some underlying latent score, modeling an abstract quantity causing the response, see Section 3. Ordinary regression analysis, however, does not allow for such an error.
(b) An ordinary regression approach does *not* provide probability estimates for the response levels but only a prediction of the estimated value given covariates (possibly with corresponding confidence interval to quantify uncertainty).
(c) Linear regression may yield predictions beyond the original response scale (i.e., above the highest or below the lowest level).
(d) Linear regression ignores different variabilities in the response categories: usually there is only a small variability at predictor values for which observations fall mainly in the highest (or lowest) category, but there is a considerable variability at predictor values for which observations tend to spread among the categories.

As for the presented application, see Section 1.1, the distribution of consent and the number of people responding “rather consent” was of particular interest, we argue that an ordinal analysis is the most appropriate in this case. For further applications and discussions about model choices we refer to Agresti [2, section 1.3] and the references therein.

## 3 Ordinal Regression Models

In ordinal statistics, the quantity of interest is typically an ordinal response (e.g., of a survey), modeled by a random variable Y which is assumed to take values on an *ordinal scale* with L≥2 levels, 1≤2≤⋯≤L, (e.g., the levels of consent). Note that although the levels are encoded numerically, it is neither assumed that we can interpret between‐level distances (e.g., 2−1) nor that the distances between two levels are equidistant—there might be a large difference between “unsure” and “rather consent” but only a small one between “rather consent” and “absolutely consent.”

### 3.1 Latent Variable Approach

**FIGURE 2:**
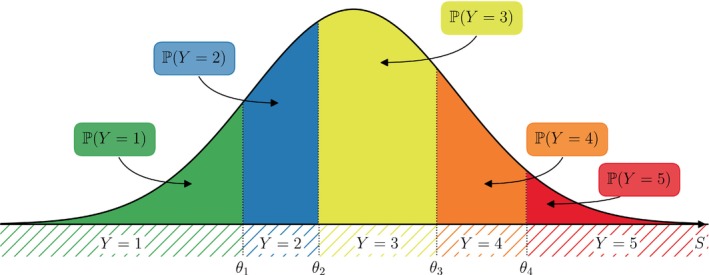
Probability distribution of the response Y (hatched regions) to a questionnaire with L=5 possible answers (encoded as 1 to 5). The response probabilities are acquired as the area (colored regions) under the density curve of S between two consecutive cut points $\theta_{\ell },\theta_{\ell +1}$, ℓ=0,1,2,3,4.

For ordinal regression, we assume that Y can be acquired as the discretization of an unobserved, continuous *latent score* S as

(1) $$Y=\ell \;\;\text{if and only if}\;\theta_{\ell -1}<S\leq \theta_{\ell }$$

for all levels ℓ=1,2,…,L where the $\theta_{\ell }$'s are *cut‐points* (also *threshold coefficients*) corresponding to the level boundaries on the scale of the latent variable, see Figure 2. The cut‐points $\theta_{\ell }$ are assumed to be ordered in a strictly increasing manner $-\infty =\theta_{0}<\theta_{1}<\cdots <\theta_{L-1}<\theta_{L}=+\infty$ and have to be estimated within a regression framework. For an ordinal variable with L levels we have to estimate L−1 cut‐points $\theta_{1},\theta_{2},\ldots ,\theta_{L-1}$. Of note, the term *cut‐points* is a traditional term for an endpoint in one inequality of (1); it is not meant to suggest cutting data and is not related to categorization or loss of information.

The latent variable includes all external parameters which can influence the response behavior of the responder and is often thought of as an abstract quantity, for example, consent, quality of life or pain, of which Y=1,2,…,L represent the ordinal levels for example, “absolutely consent”, “rather consent”, “unsure”, “rather decline”, “absolutely decline” (for L=5).

From Equation (1) follows that the probability distributions of Y and S are related as $\mathbb{P}(Y\leq \ell )=\mathbb{P}(S\leq \theta_{\ell })$ and in particular $\mathbb{P}(Y=\ell )=\mathbb{P}(\theta_{\ell -1}<S\leq \theta_{\ell })=\mathbb{P}(S\leq \theta_{\ell })-\mathbb{P}(S\leq \theta_{\ell -1})$. Within an ordinal regression framework, the influence of the covariates $\text{x}={\left(x_{1},x_{2},\ldots ,x_{K}\right)}^{⊤}\in {\mathbb{R}}^{K}$ on the response Y is typically modeled on the latent scale as

(2) $$S={\text{x}}^{⊤}\beta +\varepsilon =\overset{K}{\underset{k=1}{\sum }}x_{k}\beta_{k}+\varepsilon$$

where $\beta ={\left(\beta_{1},\beta_{2},\ldots ,\beta_{K}\right)}^{⊤}\in {\mathbb{R}}^{K}$ contains the regression parameters and ε is a random variable whose distribution needs to be specified, typically with zero mean and known variance. Some common choices are stated below in Section 3.2.1.

### 3.2 Methodological Details

#### 3.2.1 Link Functions

(a) Assume ε∼𝒩(0,1) to be standard normally distributed. Then, we obtain the probability distribution of the survey response Y given covariates x via S as
(3) $$\begin{array}{ll} \mathbb{P}(Y\leq \ell |\text{x}) & =\mathbb{P}\left(S\leq \theta_{\ell }|\text{x}\right)=\mathbb{P}\left(S-{\text{x}}^{⊤}\beta \leq \theta_{\ell }-{\text{x}}^{⊤}\beta |\text{x}\right)\\ & \overset{\left(2\right)}{=}\Phi \left(\theta_{\ell }-{\text{x}}^{⊤}\beta \right) \end{array}$$
  where Φ is the distribution function of the standard normal distribution. Particularly, the probability for a response answer can be obtained as the area under a normal curve between two consecutive cut points (possibly shifted by the term including information about covariates), see Figure 2. This model is, in relation to the probit model for binary responses, also called *ordered probit* model.
(b) Another popular choice is to assume ε to follow a standard logistic distribution, that is, ε has cumulative distribution function $F(t)=\mathbb{P}(\varepsilon \leq t)=1/(1+e^{-t})$. Then, similarly to Equation (3), we obtain
$$\mathbb{P}(Y\leq \ell |\text{x})=F\left(\theta_{\ell }-{\text{x}}^{⊤}\beta \right)=\frac{1}{1+e^{-\left(\theta_{\ell }-{\text{x}}^{⊤}\beta \right)}}$$
  and for the log‐odds holds
(4) $$\log\left(\frac{\mathbb{P}(Y\leq \ell |\text{x})}{1-\mathbb{P}(Y\leq \ell |\text{x})}\right)=\theta_{\ell }-{\text{x}}^{⊤}\beta ,\;\ell =1,2,\ldots ,L$$
  Thus, this model is also called *proportional odds* or *ordered logistic regression* model [6, 7]. Note that for L=2, that is, for binary responses, this model reduces to the ordinary logistic regression model.
(c) More generally, S can be modeled to follow an arbitrary distribution. Christensen [13, section 2.2] provide a comprehensive overview about the choice of common link functions beyond the ones presented above, depending on the assumptions of the rating behavior of the responder, for example, choose a Cauchy distribution if extreme ratings are assumed to be more likely. Moreover, Agresti [2, chapter 5] yield a differentiated view on the field of ordinal models with various examples and highlight aspects about practical and theoretical issues.

#### 3.2.2 Cumulative Link Models

From Equation (3) follows (for an arbitrary choice of ε) that we can write the cumulative distribution function (CDF) of Y given the covariates x as

(5) $$\mathbb{P}(Y\leq \ell |\text{x})=F(\theta_{\ell }-{\text{x}}^{⊤}\beta ),\;\ell =1,2,\ldots ,L$$

where F is the distribution function of ε. As the function F links the CDFs of Y and S, ordinal models of the form (5) are called *cumulative link models* and F is called *inverse link function* (as it takes the linear predictor $\theta_{\ell }-{\text{x}}^{⊤}\beta$ from the latent space back and maps it to predicted probabilities for Y) [13].

#### 3.2.3 Intercept

Note, that in contrast to ordinary linear regression, the model from Equation (2) deliberately does *not* include an intercept term, as a model with $S=\alpha +{\text{x}}^{⊤}\beta +\varepsilon$ and shifted cut points ${\tilde{\theta }}_{\ell }=\theta_{\ell }-\alpha$ would result in the same distribution for the response Y. Thus, the cut‐points $\theta_{\ell }$ would not be identifiable in a model including a fixed *unknown* intercept. A model including a fixed *known* intercept, however, would result in the same parameter estimates β as for the model (2). For this reason, without loss of generality α=0 is assumed.

### 3.3 Interpretation of Model Parameters

To conclude this section, let us briefly address the influence of the regression coefficients. An interpretation of this influence on the response variable Y is, in general, difficult and depends on the chosen link function, compare Agresti [2, section 5.1.3]. For the logit‐link, there is the following relation for the difference in log‐odds (i.e., the logarithm of the odds ratio). Note that here, the odds are the ratio of responding at most ℓ vs. responding at least ℓ+1, ℓ=1,2,…,L. Denote the vector of covariates by $\text{x}=(x_{1},x_{2},\ldots ,x_{K})$ and let $\tilde{\text{x}}=(x_{1},\ldots ,x_{k-1},x_{k}+1,x_{k+1},\ldots ,x_{K})$ be differing from x only at the k‐th component by one, then

$$\begin{array}{rl} \log\left(\frac{\mathbb{P}(Y\leq \ell |\text{x})}{1-\mathbb{P}(Y\leq \ell |\text{x})}\right)-\log\left(\frac{\mathbb{P}(Y\leq \ell |\tilde{\text{x}})}{1-\mathbb{P}(Y\leq \ell |\tilde{\text{x}})}\right) & \overset{\left(4\right)}{=}\theta_{\ell }-{\text{x}}^{⊤}\beta -\left(\theta_{\ell }-{\tilde{\text{x}}}^{⊤}\beta \right)\\ & ={\left(\tilde{\text{x}}-\text{x}\right)}^{⊤}\beta =\beta_{k} \end{array}$$

for all ℓ=1,2,…,L.

Thus, for a binary group variable, the coefficient $\beta_{k}$ is the change in the log‐odds for a change in groups for each cumulative probability, keeping all other parameters fixed. More general, for a continuous covariate $x_{k}$, $\beta_{k}$ is the effect of a *unit increase* in $x_{k}$ on the log‐odds for each cumulative probability, controlling for all the other predictors. As a result, $\exp(\beta_{k})$ is a cumulative odds ratio using any collapsing of the ordinal response for values of $x_{k}$ that differ by 1 unit. The corresponding consequences for the question analyzed and an interpretation beyond that are, however, often debatable. Here, the interpretation of predicted response probabilities is more recommended and represents the true strength of ordinal models.

For an arbitrary distribution of S, we can still claim that a unit increase in $x_{k}$ corresponds to an increase in the mean 𝔼S of S by $\beta_{k}$, keeping the other predictor values fixed, compare Agresti [2, sections 5.1.3 and 5.2.1]. Stating a relation between the regression parameters and the response distribution in general is, however, not possible.

## 4 Identification of Influencing Factors and Effect Quantification

**FIGURE 3:**
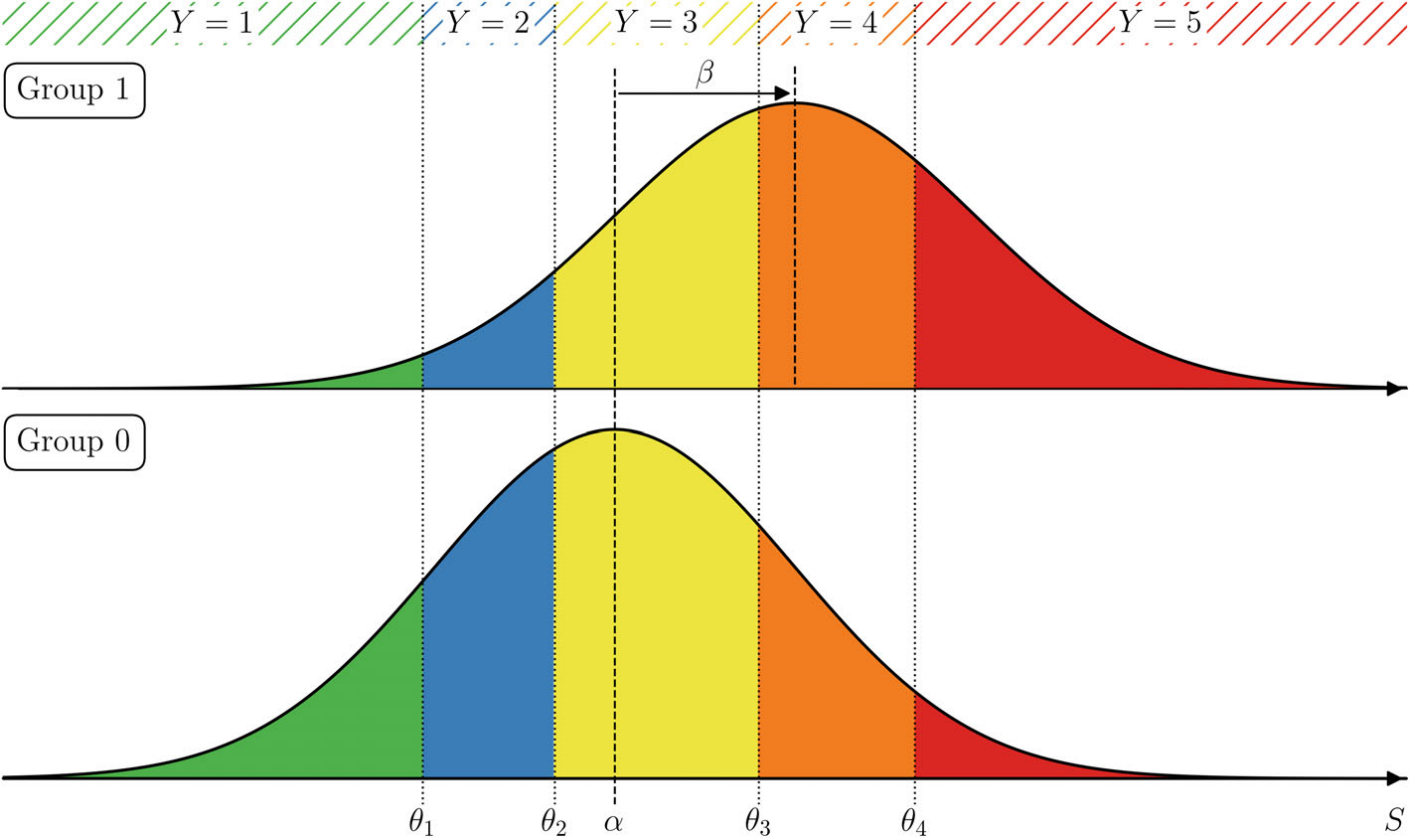
Graphical representation of the influence of a binary group variable on the distribution of the latent variable S and effects on the probabilities of the survey responses Y: The entire distribution of the latent variable, particularly its mean α (dashed lines), is shifted by the regression coefficient β to the right (left) if β>0 (β<0). The probabilities (colored areas) for all response levels change accordingly.

Let us now investigate the influence of covariates on the response outcome. To this end, we consider at first discrete covariates, say, a binary group variable having two levels 0/1 (e.g., two different studies with one question each having the same possible answers). Then, the distribution of the latent variable S for group 1 is the one for group 0 shifted by the regression coefficient β, see Figure 3. This results in a shift of the probability masses for all possible responses. In the example, this means that positive values for β make responses encoded with high values more likely (and responses encoded with low values less likely), and vice versa for negative values of β.

Using the framework presented above, we are moreover able to test for significant differences in the rating behavior between two groups. Two rating behaviors are considered to differ significantly if the corresponding regression coefficient β on the latent scale differs significantly from zero, that is, there is a significant shift in the distribution of the latent variable S. Testing procedures are of asymptotic Wald‐type, compare Christensen [12, section 4.1].

### 4.1 Model Application

We consider the data from the filippa study from Section 1.1. We are interested whether and how the complexity of the study (observational/ RCT/ pharmacological study) and the child's sex influence the response behavior of the legal representatives. Therefore, we fit an ordinal regression model for the response with covariates study complexity and child's sex with logit‐link using the clm function from the ordinal package:

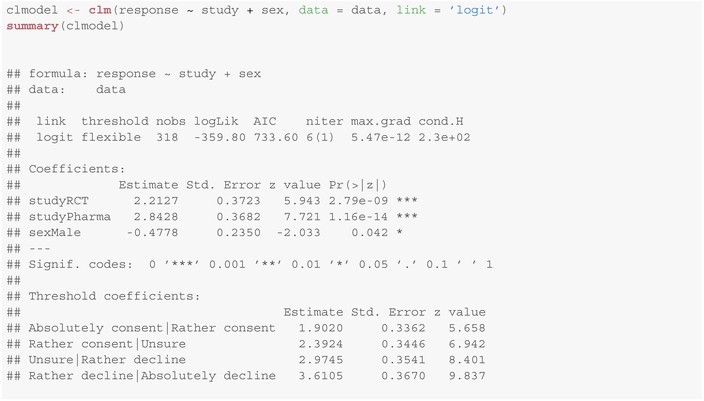

This model provides:

- L−1=4 threshold coefficients (cut‐points), corresponding to the $\theta_{\ell }$'s in Figure 3;
- two coefficients for the levels “RCT” (studyRCT) and “pharmacological study” (studyPharma) of complexity (in comparison to the “observational study”);
- and one coefficient sexMale for the influence of the child's sex (here: boys in comparison to girls).

The estimated coefficients for study complexity and sex are statistically significant (according to a Wald test, p<0.001 and p=0.042, respectively) at the two‐sided 5% significance level. As the coefficient for study complexity “RCT” is positive, the latent score distribution of Y for an RCT study is shifted to the right by 2.2127 in comparison to the observational study. See Figure 3 for an illustration where 1 denotes the highest level of consent (“absolutely consent”) and 5 the least (“absolutely decline”). As the cut‐points do not depend on the covariates, by this shift we predict levels with lesser consent with higher probability.

A similar argument holds for the pharmacological study compared to the observational study (here, the shift is 2.8428, that is, another 0.6301 units with respect to “RCT”). Moreover, as the coefficient for sex is negative (−0.4778), legal representatives of boys were significantly more likely to consent to participate in the studies than those of girls (p=0.042).

To assess whether there is also a significantly different response behavior between the RCT and the pharmacological study, we have to conduct one additional test. Note that to this, we have to adjust for multiple testing. For this purpose, the emmeans method from the package of the same name emmeans [26] provides several possibilities using the adjust parameter. By default, p‐value adjustment using Tukey's method [32], for multiple comparisons is applied:

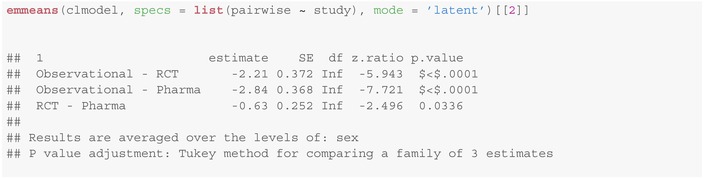

See also [33, section 5] for the theoretical background and a comprehensive overview about multiple testing problems. From the last column it follows that there are significant differences in the response behavior of voters between all three study types at the significance level 5%.

Note that in the model summary there are no p‐values provided for the threshold coefficients as testing against zero would not make much sense—the actual position of the cut‐points has no meaning but only the distances between each other and the relative position to the mean of the latent variable, compare Section 3.2.3.

For this study, the odds for a legal representative of a girl to respond “absolutely consent” for the observational study are about exp(2.2127)=9.1 times the odds for the RCT. This estimate is, however, relatively imprecise which can probably be reduced to the small number of legal representatives of a girl absolutely willing to participate in the RCT or the small sample size of the study overall. The 95%‐confidence interval is [1.5154,2.9858], corresponding to [4.551,19.803] for the odds ratio. As descriptive statistics of effects (here: probabilities) can compare the cumulative probabilities more suitably and are easier to interpret, we recommend to describe effects for ordinal regression quantitatively by presenting comparisons of probabilities for example, at their extreme values, see also Agresti [2, section 8.2.4]. The procedure how to do so is described as follows.

### 4.2 Predicting Probabilities, Confidence Intervals

The package emmeans provides moreover a method for computing predicted marginal response probabilities for each possible answer (columns response and prob), possibly stratified by given covariates (first line in each paragraph below):

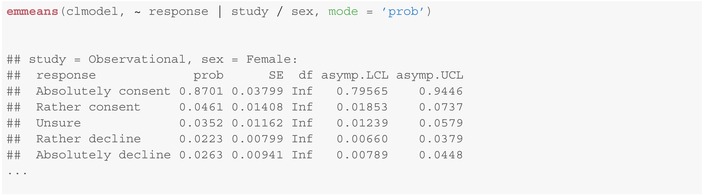

Note, that as the regression parameters and cut‐points are subject to uncertainties (the data is random), so are the *estimated probabilities* of the possible responses. Corresponding asymptotic (indicated by the infinite number of degrees of freedom df=Inf) confidence intervals, given as [ asymp.LCL, asymp.UCL ], can be derived using the delta method and are provided in addition to the predictions in the emmeans method above (last two columns). We refer to Christensen [13, section 4.7] for further details on the derivation of standard errors (column SE) to compute these confidence intervals.

The emmeans package contains moreover a function to show the regression output graphically using emmip for a clear and compact presentation:

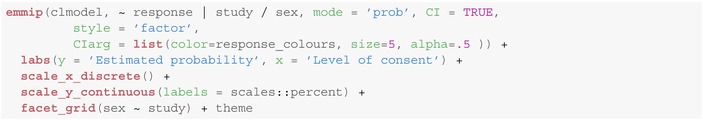

**FIGURE 4:**
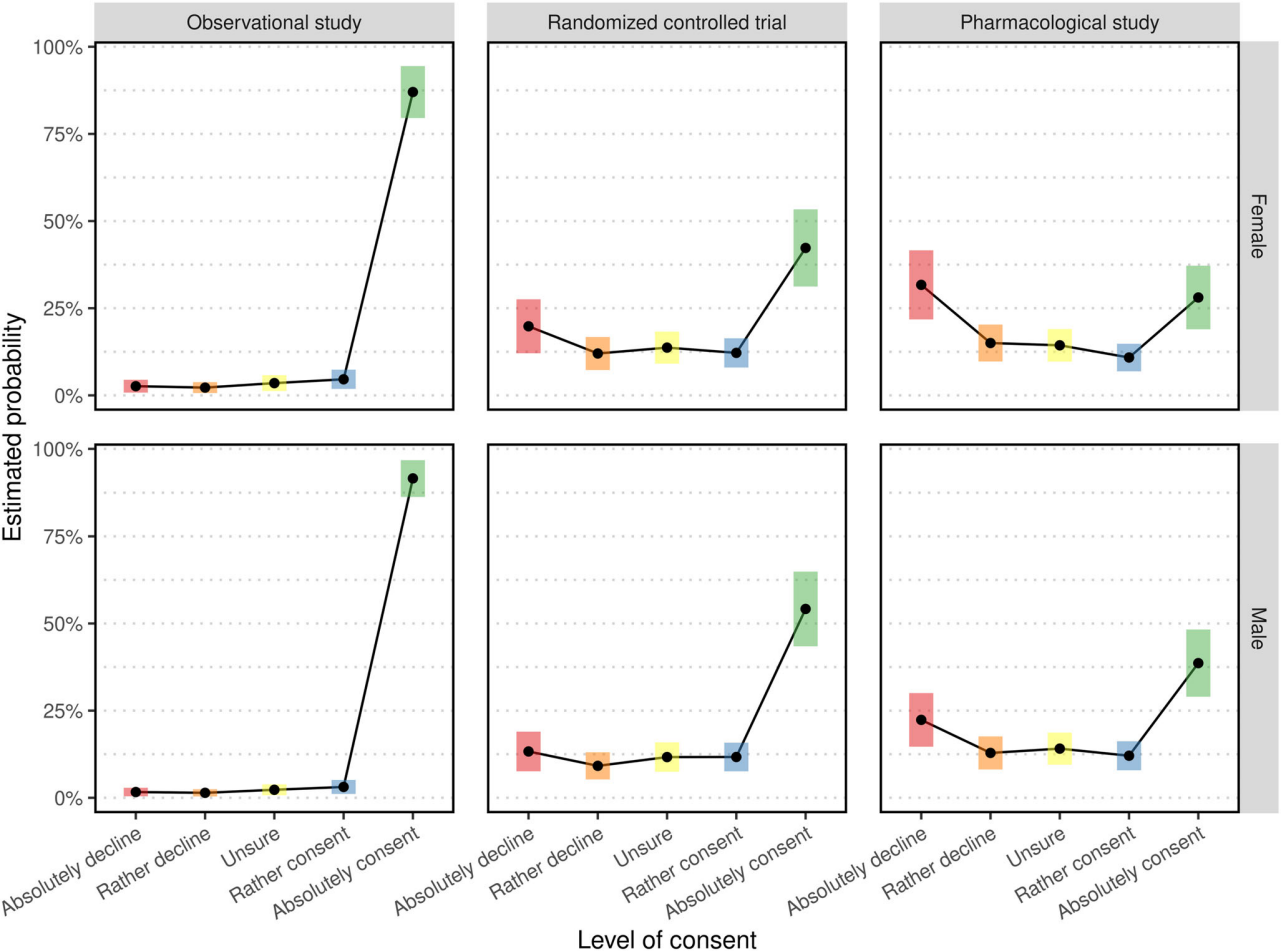
Estimated response probabilities stratified by study complexity (horizontally) and child's sex (vertically) within the ordinal regression model of Section 4.1.

Figure 4 clearly shows differences in the response behavior of legal representatives depending on the study complexity. More precisely, the statement that willingness to consent to participation decreases with increasing level of complexity from Section 4.1 is confirmed. Further, we discern smaller differences between the willingness to consent between boys and girls. Particularly for the RCT and the pharmacological study, more participants were responding “absolutely consent” and less “absolutely decline” in the boys' strata than in girls'. Differences were about 13% and 6% for the RCT and about 11% and 10% for the pharmacological study, respectively.

Finally we like to remark that emmeans provides for the possibility to compute cumulative and exceedance (i.e., 1 − cumulative) probabilities using mode = 'cum.prob' and mode = 'exc.prob', respectively. For instance, the probability for a legal representative of at least “rather consenting” is:

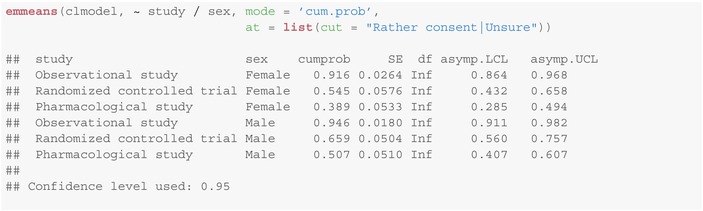

Note, that the cumulative and exceedance probabilities can be obtained from the individual probability estimates above. This, however, does not hold for the confidence intervals.

For the model parameters, the ordinal package provides moreover profile likelihood based confidence intervals for model covariates. These confidence intervals often possess better coverage than Wald‐type confidence intervals, particularly in studies with small to moderate sample size. The computation can be performed via:

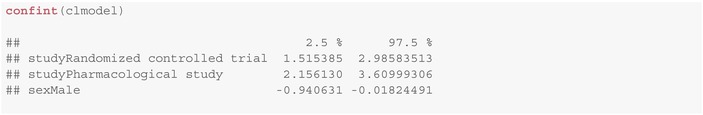

Profile likelihood based confidence intervals are implemented for regression and scale parameters, but are not available for threshold, nominal and flexible link parameters [13].

### 4.3 Numerics and Convergence

The model coefficients are estimated numerically using a maximum likelihood approach by calculation of zeroes of the gradient of the negative log‐likelihood. Parameter estimates are output after a convergence criterion is satisfied (typically small gradient) or a maximum number of iterations has been reached. Numerics and control parameters can be passed using clm.control.

To oversee the convergence of the approach, the summary of the clm method provides three parameters, see Section 4.1: niter (the number of Newton‐Raphson iterations needed with the number of step‐halvings in parentheses), max.grad (the maximum absolute gradient of log‐likelihood) and cond.H (the condition of the Hessian at the maximum). More detailed information can be obtained using the convergence method applied to the model (see also Section 5.1 below). Christensen [12] states that large cond.H values (like > 1e4) might indicate that the model is ill‐defined. As in the case of Section 4.1 max.grad is small and cond.H is reasonably sized, we can conclude that the algorithm seems to have converged properly.

### 4.4 Interaction Effects

One might wonder, whether it is appropriate to include also an interaction term study:sex in the model. To this end, we can fit a model including this interaction and compare it with the model from Section 4.1 (not containing factor interaction). For this purpose, the ordinal package provides the anova method conducting a likelihood ratio test between both models:

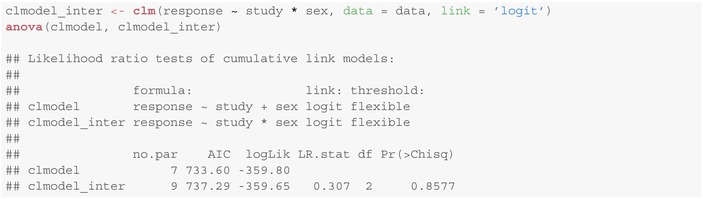

This implies that there is no statistical evidence for including interaction study:sex between study complexity and child's sex into the model (p=0.8577).

### 4.5 Random Effects

To conclude this first example, we consider the question whether there are substantial differences in the individual response behaviors, that is, whether there are parents responding systematically for example, particularly low or high values or answering always “unsure” etc. To this end, we fit an ordinal model as in Section 4.1 but with additional individual random effect using the clmm function from the ordinal package and compare this model, analogously to Section 4.4 with the model from Section 4.1:

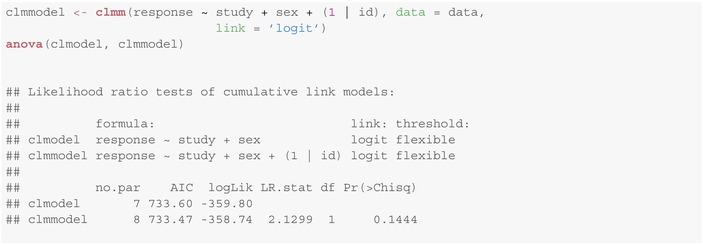

Again, there is no strong evidence (p=0.1444) for substantial subject specific response behaviors; a test at the 5% significance level would not reject the model from Section 4.1 in favor to a model including additional individual random effects.

Note that, as of now, the clmm function does not support predicting probabilities of a model containing random effects. In contrast, the former implementation clmm2 of clmm does support prediction, however, provides only fitted values for a random effect of zero [12]. Beyond this, we present an approximate approach using the emmeans method at the end of the following Section 4.6.

Of note, methods from the ordinal package seem to often not converge when there are random effects included. In this case, it might be worthwhile to consider for example, a Bayesian random effects ordinal logistic model, compare the blrm from the rmsb package [20]. Moreover, a further alternative for including repeated measures into ordinal models is implemented in the repolr package [34] which extends the polr from the MASS package [35]. More sophisticated designs for analyzing survey data often involve clustering and probability sampling weights. We refer the interested reader to An [36], Selosse, Jacques, Biernacki [37], or Agresti [2, chapter 12ff].

### 4.6 Continuous Covariates

**FIGURE 5:**
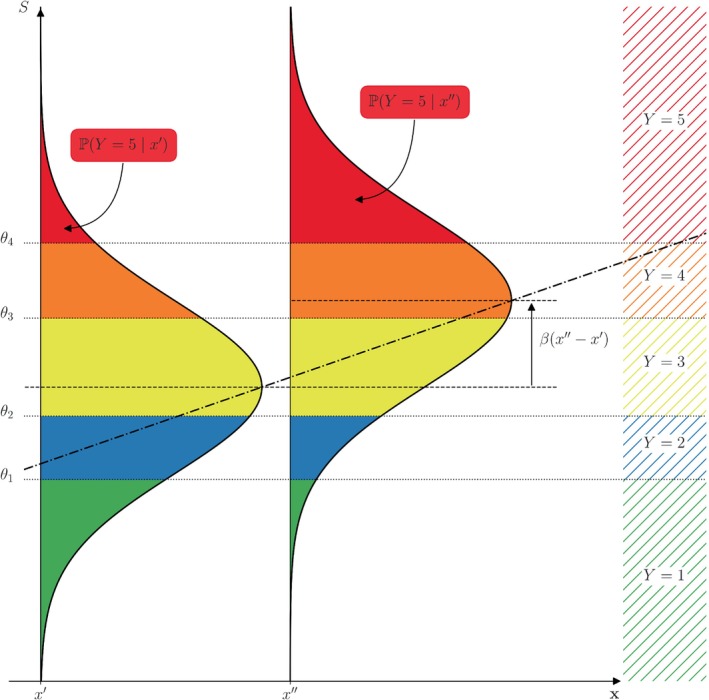
Graphical representation of the influence of a univariate continuous covariate $\text{x}\in {\mathbb{R}}^{1}$ to the distribution of the latent variable S and effects on the probabilities of the survey responses Y: A change of the covariate x causes a change of the mean of the latent score 𝔼S (cf. α in Figure 3) along the regression line (dash‐dotted −·), i.e., if the line's slope is β, a change of x by one unit results in a change of 𝔼S by β units. Particularly, assuming fixed cut‐points, if the regression coefficient β is positive (negative), a higher value of x results in tendentially higher (smaller) response values.

Considering the influence of a continuous covariate on the response outcome, fitting of models is done analogously as described in Section 4.1 above. The influence of a continuous covariate is portrayed schematically in Figure 5 for a univariate covariate $x\in {\mathbb{R}}^{1}$. The covariate x is allowed to vary along the horizontal axis whereas the latent score is plotted on the vertical axis. This corresponds to Figure 3 reflected at the 45° line. A change in x by one unit results in a shift of the latent score distribution by the corresponding regression coefficient, that is, here β units. More generally, the mean of the latent score between two responders with covariates $x^{\prime }$ and $x^{\prime \prime }$ is shifted by $\pm \beta (x^{\prime \prime }-x^{\prime })$ (depending on which direction one considers). The probabilities of the response categories shift accordingly, given unchanged cut‐points, see the colored areas in Figure 5.

Below you can find the result for an ordinal model for the response depending on study complexity and child's sex, and additionally the age of the legal representative for the data from the filippa study. Note that whenever there was more than one legal representative present at the interview, we chose the age of the oldest one for this analysis.

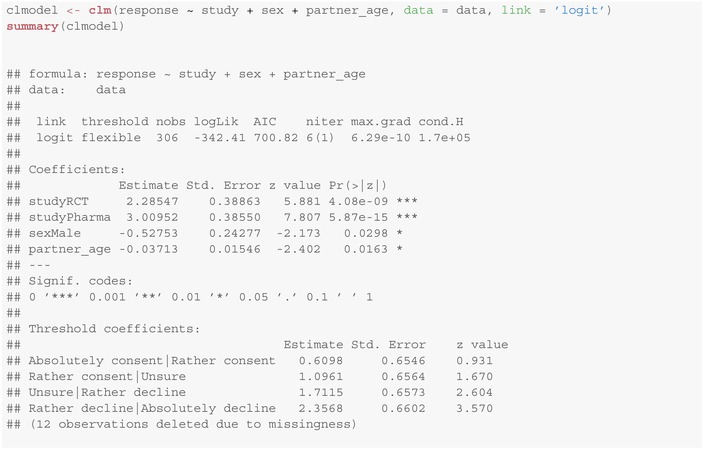

We observe that there are only minor changes in the coefficients studyRCT, studyPharma and sex in comparison to Section 4.1. The coefficient partner_age states that for every increase in the age of the legal representative by one year, the mean latent score decreases by about −0.03713 units. This means that the older the interview partners are, the more likely they are responding smaller response levels, that is, the more likely they are consenting to study participation.

The resulting matrix of estimates can then be visualized as a stream plot as follows:

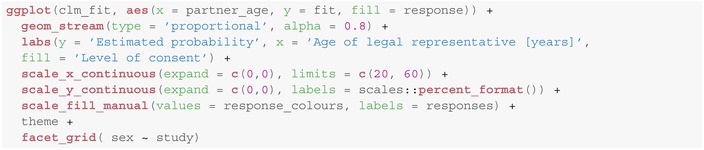

**FIGURE 6:**
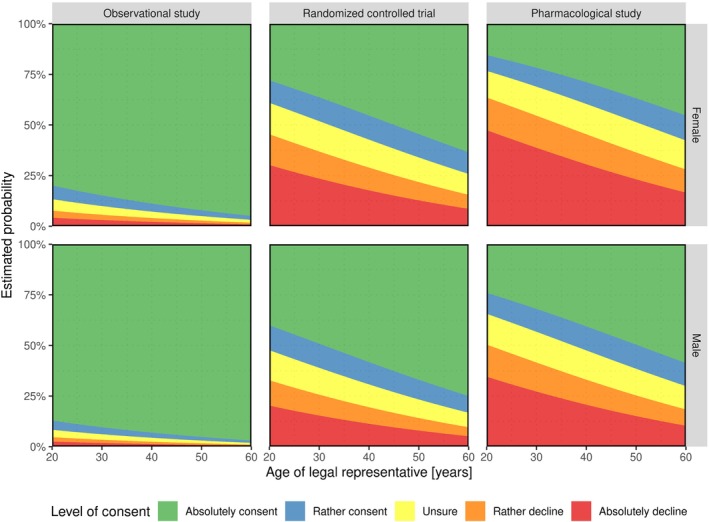
Estimated response probabilities stratified by study complexity (horizontally) and child's sex (vertically) within the ordinal regression model of Section 4.6.

In all three studies the level of consent is increasing for an increasing age of the legal representative even though this is a bit more pronounced in male than in female inpatients, see Figure 6. If we included an interaction term into the regression model, for example, study:sex between study complexity and child's sex, it might occur that this behavior differs among the different study types. However, as neither interaction between study, sex and partner_age was significant we did not include parameter interaction into the model.

Note that Figure 6, in contrast to Figure 4 does not contain any information about the estimated uncertainty of estimates (e.g., in form of confidence intervals). This is, however, rather a limitation of the graphical representation than of the applied method. We can still obtain asymptotic Wald‐type confidence intervals for the probabilities of each response level given the covariates (e.g., here for all studies and a legal representative of age 42) using:

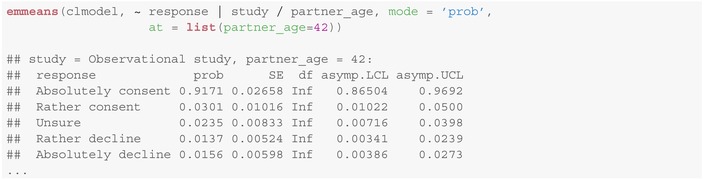

### 4.7 Invariance to Choice of Response Categories

Agresti [2, section 3.3.3] points out that the regression parameters β in the latent variable model do *not* depend on the particular way the continuous latent scale is cut by the cut‐points $\theta_{\ell }$. Thus, the effect of the parameters β are independent of the choice of the categories of Y. For instance, the same effect parameters apply for a variable with five consent levels (as in Section 4.1 and Section 4.6 above) or to a response variable with for example, ten or only three consent levels. This makes it possible to compare model parameters from studies using different scales.

## 5 Advanced Topics

To conclude we like to allude to a number of advanced topics which we deem to be important in the context of fitting ordinal models.

### 5.1 Convergence Check

As already mentioned in Section 4.3, the model coefficients are determined numerically. Whereas the summary command shows only a brief outline about model coefficients and convergence, the convergence command from the ordinal packages yields a more comprehensive overview and provides additional information about the number of correctly estimated decimals.

### 5.2 Goodness of Fit, Model Selection

In applications after model fitting typically the question for *goodness of fit* arises, that is, “How well does our model predict the present data?”. For ordinary linear regression, quantities such as an $R^{2}$ coefficient of determination, quantifying the amount of data variance that is explained by the model, are often stated. As a result, goodness of fit testing is enabled. In a nutshell, a non‐significant p‐value of a goodness of fit test indicates that there is no evidence that observed and fitted values/frequencies do differ in a statistically significant way (thus indicating a reasonable fit). Comparisons of several nested models are possible using criteria such as the Akaike (AIC) or the Bayesian information criterion (BIC) to account for increasing goodness of fit for an increasing number of included parameters, cf. Agresti [11, section 4.6].

Goodness of fit testing in ordinal models is considered by Pulkstenis and Robinson [38] and Fagerland and Hosmer [8]. Corresponding algorithms are implemented in R in the generalhoslem package [39]. For practical applications, Fagerland and Hosmer [40] recommend calculation of three different methods, the Lipsitz test, an ordinal version of the Hosmer‐Lemeshow test, and the Pulkstenis‐Robinson test, to assess goodness of fit, each covering slightly different aspects of the problem. Of note, these tests, however, do not have a good power to detect a particular type of lack of fit [5, 41]. Particularly, a large value of the global fit statistic only indicates some lack of fit, but does not provide insights about its nature [5]. For an elaborate discussion about these tests and further details we refer to Fagerland and Hosmer [40, section 6] or Hosmer Jr., Lemeshow, and Sturdivant [42, chapter 5].

Harrell Jr. [16, sections 13.3.5 and 13.3.6] proposes checking model assumptions and model fit graphically, for example, using nomograms for assessing the proportional odds assumption and quantifying the model's predictive ability using pseudo $R^{2}$ coefficients. Moreover, goodness of fit can be assessed by comparing the log‐likelihood of the model with the one of a hoped‐for‐simpler model or the one of a richer model using tests similarly to the ones presented in Sections 4.4 and 4.5.

Finally, we like to remark that there is a number of various pseudo $R^{2}$ measures and measures of predictive discrimination for ordinal models. A common variant is Nagelkerke's [43] $R^{2}$ which is also part of the standard output of the lrm function [18]. A broader variety is provided by the PseudoR2 function in the DescTool package [44]. We refer to Tjur [45] for a literature summary and some theoretical background.

### 5.3 Trial Design and Sample Size Computation

In practice, often the question arises how many participants a study should include. In case of an ordinal analysis the Hmisc package [17] provides the posamsize and popower functions to determine power of tests and sample size estimates for ordinal proportional odds models. For example, a comparison of the response behavior between study types “Observational” and “RCT” at an expected response distribution of $p={\left(0.87,0.05,0.04,0.02,0.02\right)}^{⊤}$ for the observational study and an expected odds ratio of 9, a two‐sided test on the significance level 5% achieves a power of 80% if we include 58 patients:

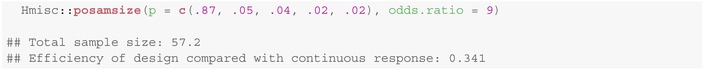

### 5.4 Model Generalizations

**FIGURE 7:**
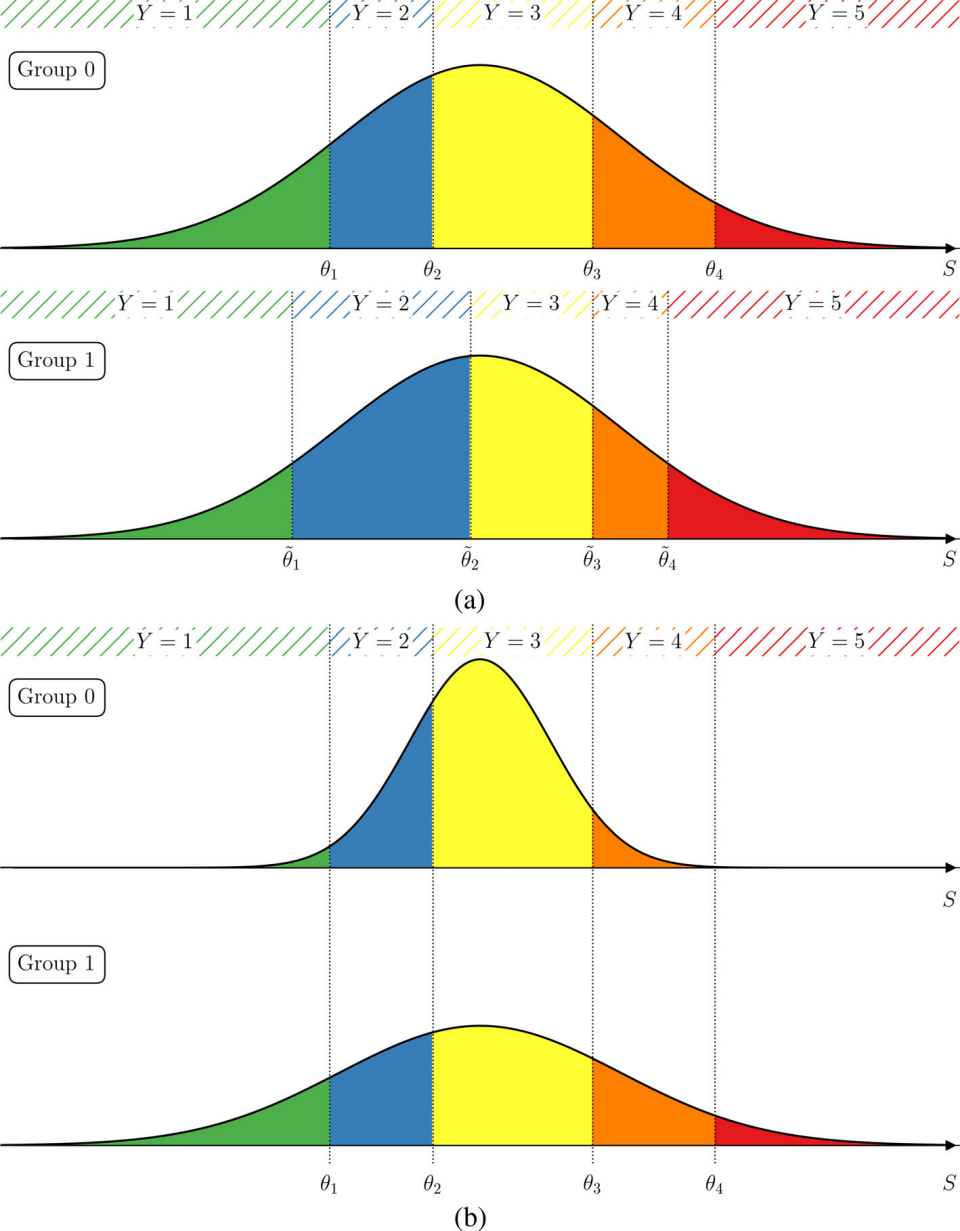
Effects of including (a) nominal and (b) scale effects to an ordinal regression model. (a) For every nominal effect we have to estimate another L (number of response levels) regression parameters, or equivalently L threshold coefficients ${\tilde{\theta }}_{\ell }=\theta_{\ell }-{\tilde{\beta }}_{\ell }$. (b) Including scale effects changes the variance of the underlying latent variable S.

Finally, the ordinal package provides a number of generalizations of the ordinal model presented in Section 3. The general form of a cumulative link model (see Equation 5) can be written as

(6) $$\mathbb{P}(Y\leq \ell |\text{x},\text{w},\text{z})=F_{\lambda }\left(\frac{g_{\alpha }(\theta_{\ell })-{\text{x}}^{⊤}\beta -{\text{w}}^{⊤}{\tilde{\beta }}_{\ell }}{\exp({\text{z}}^{⊤}\zeta )}\right)$$

where the parameters affect the model as follows:

- $F_{\lambda }$ is the inverse link function which may be parametrized by a parameter λ∈ℝ. Its inverse $F_{\lambda }^{-1}$ is also referred to as *flexible link function*.
- Cut‐points may be transformed via $g_{\alpha }(\theta_{\ell })$ to be more *structured* to reduce the number of model parameters, thus increasing efficiency of the estimators. Typical choices are assumptions of symmetric distribution of the cut‐points of around the mean or having equal distances between each other (equidistant cut‐points). Unrestricted cut‐points (corresponding to $g_{\alpha }$ to be the identity) are also referred to as *flexible*.
- ${\text{x}}^{⊤}\beta$ are the ordinary regression effects as in Equation (5).
- Regression effects (or the cut‐points, respectively) might be allowed to depend on covariates to include *nominal effects* ${\text{w}}^{⊤}{\tilde{\beta }}_{\ell }$, see Figure 7a. This allows for more flexibility in the modeling of rating behaviors. Computationally, to include nominal effects one has to pass the corresponding variable to the nominal parameter of clm. Note, however, that parameters included as nominal effects cannot be included as covariates due to identifiability reasons. Models including these nominal effects with logit‐link are also called *partial* or *non‐proportional odds* models [46].
- The variance of the latent variable might be depending on covariates via $\exp({\text{z}}^{⊤}\zeta )$ (*scale effects*, see Figure 7b), for example, reflecting different variances in the rating behavior depending on group membership. To use scale effects in the clm method, pass variable names to the scale parameter while fitting the model.

A more comprehensive overview is provided in Christensen [13, section 2.3ff] with corresponding remarks concerning implementations in Section 4 [13]. Whether it is reasonable to include any of these modifications into the model is usually up to the analyzing statistician. Models can again be compared using likelihood ratio tests as described in Sections 4.4 and 4.5, respectively. For a fitted model, likelihood ratio tests for models after adding nominal or scale effects can be performed via or scale_test(clmodel), respectively. These tests can be viewed as goodness‐of‐fit tests. In the model above, there is no statistical evidence that including nominal effects into the model would increase the model fit significantly.

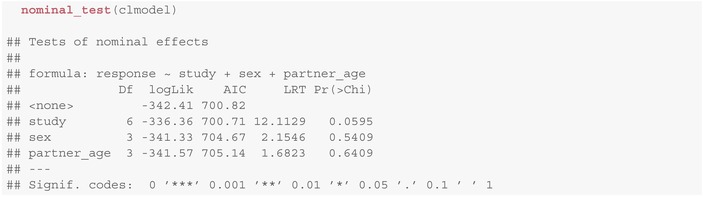

Finally, there are extensions to nonlinear ordinal regression models, that is, models in which the predictors are included in a nonlinear way. We refer for example, to the functions ordglm from the gnlm [47] package or nordr from the gln [48] package for further details.

## 6 Conclusion

The tools of the ordinal regression framework provide a method to appropriately analyze ordinally scaled data, particularly with only a few number of response categories. By applying ordinal regression we yield probabilities for each response category. This offers a more differentiated view, for example, regarding proportions of participants consenting in contrast to considering just a mean score. Moreover, considering data on a latent scale, we have the possibility of testing group differences and marginal effects such as cumulative odds. If survey data is very finely graduated, such as for numeric rating scales or comprehensive quality‐of‐life questionnaires, the usage of ordinal methods is often limited as the estimated parameters are not that meaningful anymore in contrast to, for example, a mean or median score or, are even not feasible due to a too large number of parameters to be estimated. For the filippa study, we demonstrated that a latent variable based ordinal regression analysis has the potential to identify factors influencing the willingness to consent to study participation and to quantify the probability of consenting to the participation in hypothetical studies with differing levels of complexity given a range of demographic variables from the children and their legal representatives.

## Conflicts of Interest

The authors declare no conflicts of interest. We conducted this research with institutional resources only. Open access charges were funded by the Open Access Publication Funds of the Göttingen University, who had no role in study design, data collection and analysis, decision to publish, or preparation of the manuscript.

## Supporting information


**Data S1.** Supporting Information.

## Data Availability

The raw data underlying the presented analyses will be made available by the authors upon reasonable request.
