# Supplementary material for: A Brief Introduction on Latent Variable Based Ordinal Regression Models With an Application to Survey Data
Source: Stat Med. 2024 Oct 28;43(29):5618–34. doi: 10.1002/sim.10208 (PMC11588990; doi:10.1002/sim.10208)
Supplement: Supplementary file 1 — Data S1. Supporting Information. [file SIM-43-5618-s001.pdf]

SUPPLEMENT: GGPLOT PARAMETERS

```
theme <- theme(panel.background = element_blank(),
panel.spacing = unit(1, 'lines'),
panel.border = element_rect(colour = 'black', fill=NA, linewidth=1),
axis.title.x = element_text(),
axis.title.y = element_text(),
axis.text.y = element_text(),
axis.text.x = element_text(angle = 30, vjust=1, hjust=1),
strip.text = element_text(),
panel.grid.major.x = element_blank(),
panel.grid.major.y = element_line(linewidth = 0.5,
linetype = 'dotted', colour = 'lightgrey'),
panel.grid.minor.x = element_blank(),
panel.grid.minor.y = element_line(linewidth = 0.5,
linetype = 'dotted', colour = 'lightgrey')
)
response_colours <-rep(c('#4DAF4A', '#377EB8', '#FFFF33', '#FF7F00', '#E41A1C'), 6)
```
